# Time Series Forecasting and Classification Models Based on Recurrent with Attention Mechanism and Generative Adversarial Networks

**DOI:** 10.3390/s20247211

**Published:** 2020-12-16

**Authors:** Kun Zhou, Wenyong Wang, Teng Hu, Kai Deng

**Affiliations:** 1 School of Computer Science and Engineering, University of Electronic Science and Technology of China, Chengdu 611731, China; zhoukun@std.uestc.edu.cn (K.Z.); huteng@caep.cn (T.H.); 2 Institute of Computer Application, China Academy of Engineering Physics, Mianyang 621900, China; dengkai@caep.cn

**Keywords:** time series forecasting, time series classification, long short-term memory, attention mechanism, generative adversarial network

## Abstract

Time series classification and forecasting have long been studied with the traditional statistical methods. Recently, deep learning achieved remarkable successes in areas such as image, text, video, audio processing, etc. However, research studies conducted with deep neural networks in these fields are not abundant. Therefore, in this paper, we aim to propose and evaluate several state-of-the-art neural network models in these fields. We first review the basics of representative models, namely long short-term memory and its variants, the temporal convolutional network and the generative adversarial network. Then, long short-term memory with autoencoder and attention-based models, the temporal convolutional network and the generative adversarial model are proposed and applied to time series classification and forecasting. Gaussian sliding window weights are proposed to speed the training process up. Finally, the performances of the proposed methods are assessed using five optimizers and loss functions with the public benchmark datasets, and comparisons between the proposed temporal convolutional network and several classical models are conducted. Experiments show the proposed models’ effectiveness and confirm that the temporal convolutional network is superior to long short-term memory models in sequence modeling. We conclude that the proposed temporal convolutional network reduces time consumption to around 80% compared to others while retaining the same accuracy. The unstable training process for generative adversarial network is circumvented by tuning hyperparameters and carefully choosing the appropriate optimizer of “Adam”. The proposed generative adversarial network also achieves comparable forecasting accuracy with traditional methods.

## 1. Introduction

In this paper, we focus on time series forecasting and classification tasks using state-of-the-art deep neural network (DNN) models. A time series, $X=X_{1},X_{2},\ldots ,X_{n}$, contains indexed data points in a timely order. It has been widely used in areas such as statistics, pattern recognition, communications engineering, etc. The task of time series forecasting is to establish some model based on the continuous or discrete observed values to forecast the future ones. Forecasting or prediction means transferring knowledge about samples to whole populations in statistics. Time series forecasting is different from conventional supervised learning in that the timely order should be preserved. Good models should extract information as much as possible to achieve “closest to the future” forecasting.

Time series classification aims at classifying new time series to a specific category. Suppose that there are several time series *X* in each of three categories $C_{1}=\left[X_{a},X_{a+1},\ldots \right]$, $C_{2}=\left[X_{b},X_{b+1},\ldots \right]$, $C_{3}=\left[X_{c},X_{c+1},\ldots \right]$; the task is to establish models and designate the new time series $X_{n}$ to the correct category C. A large number of machine learning classification algorithms have been proposed, such as logistic regression [1], the naive Bayes algorithm [2], support vector machines [3] and *k*-nearest neighbors [4], etc. Metrics of accuracy, precision, recall rate and receiver operating characteristic curves, etc. are used to measure the performances and trade-off between true/false positive/negative rates.

The no-free-lunch theorem states that all optimization algorithms, averaged over all optimization problems without resampling, perform equally well [5]. There is no single model that works for all given problems. Therefore, various empirical experiments have been carried out to compare performances in the specified domains. Until now, determining the best model for the specific problem was more of an art than a science.

Inspired by the recent successes of attention-based mechanisms and the temporal convolutional network (TCN) in the area of natural language processing (NLP), we proposed and applied TCN with attention to the time series forecasting and classification based on the fact that NLP and time series share sequential similarity. We also desired to verify the proposition of TCN’s superiority to the the recurrent neural network (RNN, long short-term memory (LSTM) as the representative) in sequential problems. Applying generative adversarial networks (GANs) to time series foreasting provided new insights and could potentially enhance the understanding of time series. In this contribution, we proposed LSTM with an autoencoder and attention mechanism, TCN models and a GAN model to evaluate and compare their performances for the time series forecasting and classification tasks.

### 1.1. Time Series Forecasting and Classification

Traditional statistical methods such as the autoregressive integrated moving average (ARIMA) family and exponential smoothing (ETS) were often used for time series forecasting tasks. After surveying 105 academic papers, 28 golden rules of forecasting theory were proposed in [6]. Judgement of orders of autoregressive and time lags for autoregressive moving average (ARMA). and ARIMA were summarized as best practices. Time series could be decomposed to long-term trend, seasonal, recurrent and abnormal variations with addictive or multiplicative models. CNN, RNN and the attention mechanism could be used together to avoid the weakness of CNN capturing only local dependency and promoting the strength of RNN in time series forecasting.

While deep learning has achieved huge successes in many applications, only a few time series classification algorithms have been proposed using deep NN [7]. Generative and discriminative methods have been mostly used for time series classification tasks. Autoencoder and echo state networks represent the classical generative methods. A baseline for three end-to-end algorithms, namely MLP, FCN and residual network, without imposing heavy preprocessing on the raw data, was proposed [8]. InceptionTime, a GoogleNet-like NN ensemble model, was proposed for time series classification and slightly outperformed the existing models [9]. Facebook unleashed the “prophet”, which improved the controllability and interpretability over traditional models. A comprehensive repository for research in time series classification is hosted in [10]. Fusion of deep NN models for time series represents the future direction and warrants in-depth studying.

### 1.2. Related Research

A hybrid of ARIMA and ANN models, taking advantage of the strength of ARIMA and ANN models in linear and nonlinear modeling, respectively, was proposed in [11]. A comprehensive review on forecasting methods, such as autoregressive and machine learning methods, for spatial–temporal data from a remote sensing satellite was given in [12]. The near-real-time disturbance detection method was based on least-squares spectral and cross-wavelet analyses and could be used as assessment of the results of time series [13].

Autoencoder frameworks were proposed for sequential modeling and achieved good results [14,15,16]. RNN or LSTM was used as an encoder to extract features from high-dimensional data and the decoder reconstruct dataset from feature embedding. Their results all showed that the proposed models matched or outperformed the existing methods. Research studies indicated that the dominant models for sequence tasks were mainly based on RNN or CNN with encoder-decoder architecture. Furthermore, the best performing models connect the encoder and decoder through attention mechanisms [14,15,16]. Attention mechanisms became a research hotspot and they could be applied to a variety of tasks such as machine translation, image caption generation, speech recognition, etc. Attention mechanisms improved neural machine translation (NMT) performances evidenced by BLEU (metrics of translation) scores. The combined architecture of connectionist temporal classification and LSTM (RNN) was proposed, and it achieved the best recorded score on benchmark test [17]. Transformer, the network architecture based on attention mechanisms, was proposed. Experiments presented superiority of the transformer in translation and could be potentially generalized to other sequence modeling tasks [18].

The fusion model of CNN and LightGBM to forecasting is proposed in [19]. Bias and variance for time series forecasting were investigated using the Monte Carlo method. Results suggested that ensemble NN models could potentially improve effectiveness [20]. The common research thoughts for time series classification were to extract features from time series and calculate features’ distances [21,22,23,24,25]. Their methods all outperformed other existing models under forecasting metrics.

We surveyed recent influential GAN papers and found that a majority of these papers focused on images, and that several papers focused on video generation; however, there were much fewer papers that focused on generation and time series forecasting with GAN. GAN, the epoch-making framework for estimating generative models via an adversarial process, was put forward. Experiments demonstrated the potential of the framework through qualitative and quantitative evaluation in [26]. Great development of GAN has occurred in recent years. Several hundred variants of GANs have evolved—for instance, Wasserstein GAN (WGAN) [27], SeqGAN [28], Auto-GAN [29] and stacked GAN [30], to name just a few.

WGAN improved the stability of training, eliminated mode collapse, and provided tricks useful for debugging and hyperparameter searches. Numerous papers on improving the unstable GAN training process [31,32,33] and avoiding convergence failure [34,35] were published, and these problems were still open as of the writing of this paper. Applying GANs to NLP is rather limited due to the difficulty of backpropagation through discrete random variables plus the instability of the GAN training. The authors analyzed distributions that were not differentiable, which caused problems, and they proposed their solutions in [36,37].

Traditionally, RNNs were fit for sequence modeling; however, a recent study suggested that convolutional architectures outperformed them on tasks such as audio synthesis and machine translation. A systematic evaluation of the two architectures was conducted. Experiments showed that convolutional architecture outperformed recurrent networks across different tasks and datasets. The highlight of the paper suggested replacing RNN with CNN to model sequence tasks [38].

Inspired by these up-to-date research works, we proposed the related models to empirically verify the attention mechanism’s superiority over RNN and applied GAN for the time series forecasting task.

### 1.3. Pros and Cons of the Models

RNN addressed the limitation of models having no memory, which is much needed for sequential data. LSTM extends RNN’s capability by adding long short-term memory since word’s meaning depends on the context. LSTM models were the mainstream technique for translations in the past and could also be used for text generation. Early successes and wide applications accelerated RNN’s development. However, RNN is time-consuming and deep NN cannot proceed due to its property of one word/character at a time. The shortcomings of RNN block parallel processing, while TCN has no such limits, which roughly explains TCN’s superiority to RNN in terms of efficiency.

Intuitively, CNNs excel at processing image data with geometric attributes. However, it is not ideal for sequence problems because of the filter size limit and failure of capturing long dependence information. Although CNNs are much less sequential than RNNs, the number of steps required to combine information from distant parts of the input still grows with increasing distance.

TCNs have the advantages of parallelism, low memory requirements for training and flexible receptive field size. They outperformed LSTM architectures on a variety of tasks. This architecture can map a sequence of any length to an output sequence of the same length and have longer memory than RNN. The disadvantages of TCN adaptiveness in transfer learning and the amount of history information might limit its development. Recent research boldly claimed that RNN could be even replaced by TCN, which motivates us to verify this proposition.

GAN models have evolved into several hundred variants since its inception. However, the unstable training processes and the lack of evaluation metrics remained unresolved. Although the GAN framework has remarkable performances in area of image, video, etc., the number of academic papers applying GAN to the area of time series forecasting and classification is still limited.

The paper’s contributions are summarized as follows:

1. LSTM with autoencoder and with attention mechanism is proposed and applied to the time series forecast modeling. Gaussian sliding window is proposed for the weights initialization in LSTM attention model;
2. Performances of TCN-based NN to model for time series forecasting and classification are evaluated. The proposed models outperform the classification methods such as 1NN-DTW, BOSS and WEASEL;
3. A GAN model with LSTM as the G and MLP as the D for time series forecasting task is proposed and the performances are evaluated. Comparisons are made with the statistical ARIMA models.

The organization of the paper is as follows. In Section 1, we briefly introduce the background information and related research studies. Methodologies are presented in Section 2. Proposed models are tested and results are given in Section 3. Conclusions and possible future developments are discussed in Section 4 and Section 5, respectively.

## 2. Methodology

### 2.1. Basics for the Proposed Models

**Figure 1:**
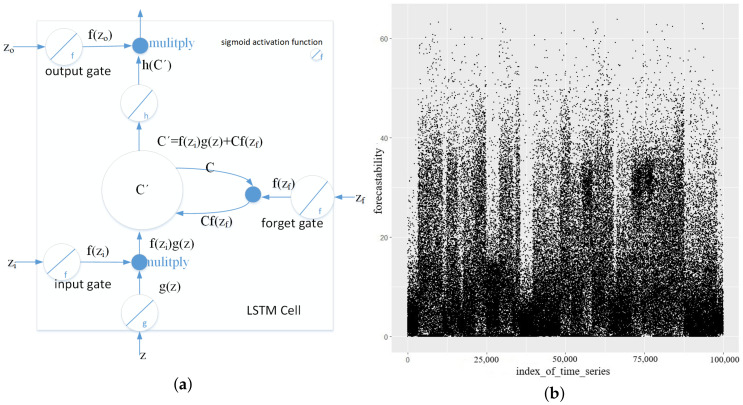
(**a**): Basic LSTM cell. (**b**): Overall Kaggle web traffic dataset forecastability.

RNN/LSTM models were used for time series forecasting modeling in the past due to its recurrent and autoregressive structure. The basic LSTM cell [39] is illustrated in Figure 1a and Equation (1). The LSTM cells are building blocks of the input layer, hidden layer and output layer of the deep NN. Autoencoders employ the unsupervised learning method to extract features from high-dimensional data and to reconstruct them from feature representations. They consist of encoding and decoding processes, where encoding maps the input to the feature spaces and then decodes back to the original spaces.

$$o_{t}=\sigma (W_{o}\cdot \left[h_{t-1},x_{t}+b_{o}\right]\;\left(output\;gate\right)$$

$$h_{t}=o_{t}*\tanh\left(C_{t}\right)$$

$$C_{t}=f_{t}*C_{t-1}+i_{t}*\tilde{C_{t}}\;\left(new\;state\;C_{t}\right)$$

$$\tilde{C_{t}}=\tanh\left(W_{c}\cdot \left[h_{t-1},x_{t}\right]+b_{c}\right)\;\left(candidate\;state\;\tilde{C_{t}}\right)$$

$$i_{t}=\sigma \left(w_{i}\cdot \left[h_{t-1},x_{t}\right]+b_{i}\right)\;\left(input\;gate\right)$$

(1) $$f_{t}=\sigma \left(w_{f}\cdot \left[h_{t-1},x_{t}\right]+b_{f}\right)\;\left(forget\;gate\right)\;\;$$

**Figure 2:**
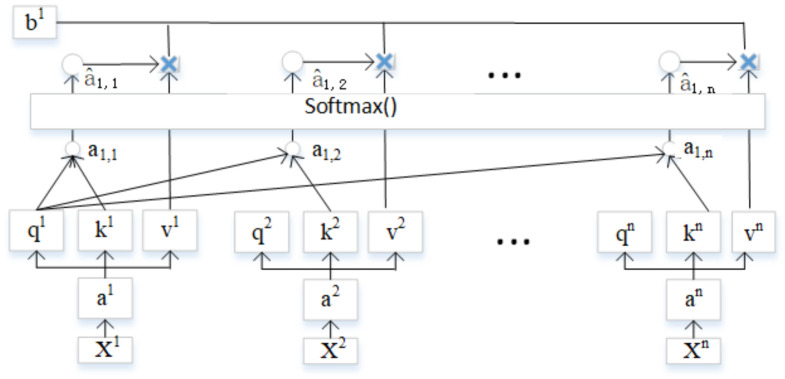
Basic attention mechanism.

Recent study suggested that RNNs could be replaced by models such as attention-based NN. In practice, LSTM-based neural machine translation (NMT) has been replaced by Google, Salesforce, etc. Wave-net proposed by Google for speech synthesis and CNN-based models for machine translation by Facebook outperformed other models. The additive and multiplicative attention functions were introduced in [18]. The attention mechanisms map vectors input (query, key, values) to a vector output, which is computed as a weighted sum. The scaled dot-product attention detailed on the computation of attention function where queries were packed into a matrix Q, keys and values into matrices K and V. Query vector $q^{i}=w^{q}a^{i}$, thus ($q^{1},q^{2},\ldots ,q^{n}$) = $w^{q}$($a^{1},a^{2},\ldots ,a^{n}$), Key vector $k^{i}$ = $w^{k}a^{i}$ ($k^{1},k^{2},\ldots ,k^{n}$) = $w^{k}$($a^{1},a^{2},\ldots ,a^{n}$), and Value vector $v^{i}=w^{v}a^{i}$, ($v^{1},v^{2},\ldots ,v^{n}$) = $w^{v}$($a^{1},a^{2},\ldots ,a^{n}$), $a_{1,1}=k^{1}q^{1}$, $a_{1,2}=k^{2}q^{1}$… We gave the *A* matrix computation (the computation for *K* and *V* were similar) in Equation (2) and Figure 2. $Attention\left(Q,K,V\right)=softmax\left(\frac{QK^{T}}{\sqrt{d_{k}}}\right)V$.

(2) $$A=\left[\begin{array}{ccc} a_{1,1} & a_{2,1}\ldots & a_{n,1}\\ a_{1,2} & a_{2,2}\ldots & a_{n,2}\\ \ldots & & \\ a_{n,1} & a_{n,1}\ldots & a_{n,n} \end{array}\right]=\left[\begin{array}{c} k^{1}\\ k^{2}\\ \ldots \\ k^{n} \end{array}\right]\left[\begin{array}{ccc} q^{1} & q^{2}\ldots & q^{n} \end{array}\right]=K^{T}Q$$

The TCN model combining dilated and causal convolutional layers that was presented ensured that the previous time step will not use future information because the output of time step $T_{t}$ was obtained based on $T_{t-1}$. TCN can be viewed as 1D FCN plus one-dimensional causal convolutions, and each two convolutional layers and identity mapping are encapsulated into a residual module. The residual module then stacks the deep network, and uses fully convolutional layers in the last few layers. Dilated convolution enlarges the receptive field exponentially [38]. We simplified the TCN model’s highlights using Equation (3). Dilated convolution operation F on elements of the sequence X is defined as:

(3) $$F\left(s\right)=\left(X{*}_{d}f\right)\left(s\right)=\sum_{i=0}^{k-1}f\left(i\right)\cdot X_{s-d\cdot i}\;$$

where $X\in R^{n},f:\left\{0,\ldots k-1\right\}\to R$, d stands for dilation rate and f for filter size. For the GAN structure, there are two NNs, Generator (*G*) and Discriminator (*D*). *G* and *D* contest with each other in which *G* generates candidates while *D* evaluates them. *G*’s training objective is to increase the error rate of *D*. The training could be expressed by the classical value function [26] demonstrated in Equation (4).

(4) $$\underset{G}{Min}\underset{D}{Max}=E_{x∼p_{data}\left(x\right)}\left[logD\left(x\right)\right]+E_{z∼p_{z}\left(z\right)}\left[log\left(1-D\left(G\left(z\right)\right)\right)\right]\;$$

where *G*’s distribution is represented by $p_{g}$ over data *x*, a priori on input noise variables is $p_{z}\left(z\right)$, *G* is a differentiable function represented by a multilayer perceptron (MLP) and *D*(*x*) represents the probability that *x* comes from the data rather than $p_{g}$. The maximizing of assigning correct labels for *D* and minimizing of log(1−D(G(z)) are simultaneously trained.

### 2.2. Datasets

Public datasets were collected for the proposed models. Kaggle web traffic competition was used for the time series forecasting task [40]. We evaluated the performance of the proposed LSTM, TCN and GAN models using this dataset. ECG, a multivariate and classified dataset, was used to test the performance of time series classification task with the TCN model. The dataset has 19 features, 150 time steps and 20 classes—that is, 19 features were collected for 150 time steps and they were classified into 20 classes. The VPN-nonVPN dataset from the Canadian Institute of Cybersecurity was used for time series classification, which can be found at [41].

The adding problem has been used repeatedly as a stress test for sequence models [39]. MNIST images are presented to the model as a 784 * 1 sequence for digit classification [42]. The memory-copying task is to generate an output of the same length as the input sequence with predefined restraints [43].

The dataset should generally be preprocessed before entry into the models. Typical preprocessing consists of center and scale, impute for missing value, the dealing of imbalanced classification problems (many more samples for one class and less for other), or the wrong labels, etc. There was some missing value for the whole web traffic dataset and we deleted those missing value datasets and used 100,000 for the tests. The ECG dataset was perfectly labeled and no extra preprocessing was needed, and for the VPN-nonVPN dataset, the scaling and centering was used.

The forecastability computation was defined using spectral density in Equation (5). We calculated the forecastability of the Kaggle web traffic dataset of 100,000 time series and plot them in Figure 1b. Each dot in Figure 1b represents one time series’ forecastability. The larger value of forecastability, the easier to forecast. For example, the value of white noise nearly reaches 0, which means it’s hard to forecast. We obtained the whole picture of dataset forecastability from reading Figure 1b.

(5) $$\omega \left(x_{t}\right)=1+\frac{\int_{-\pi }^{\pi }f_{x}\left(\theta \right)logf_{x}\left(\theta \right)d\theta }{log2\pi }$$

where $x_{t}$ stands for stationary process, $f_{x}\left(\theta \right)$ for normalized spectral density of the time series. In particular $\int_{-\pi }^{\pi }f_{x}\left(\lambda \right)d\lambda =1$.

### 2.3. Long Short-Term Memory with Autoencoder and Attention

**Figure 3:**
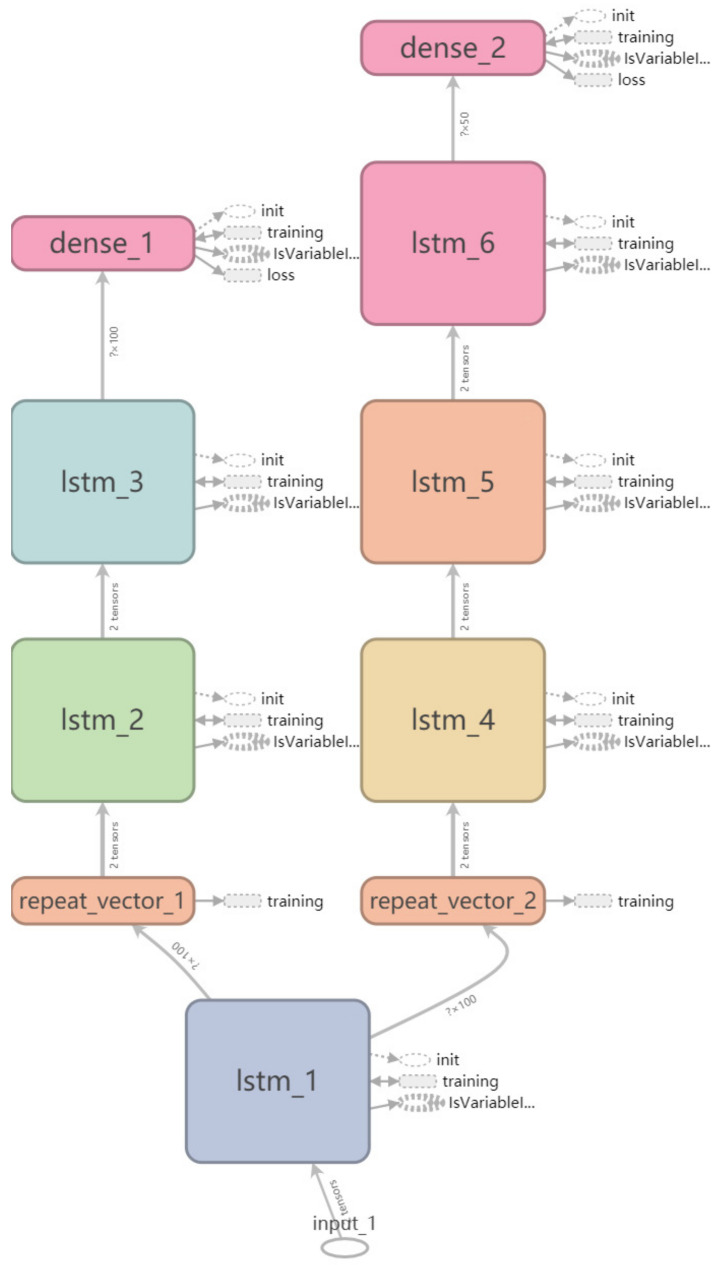
Architecture for LSTM with autoencoder.

The LSTM with autoencoder was proposed to forecast the web traffic. The input was preprocessed as time series data and the output were the 500 training and the forecasting horizon of 50 steps forward (90% of the dataset was used for training and the remaining for test). We deleted the time series with missing values. We designed one encoder and one decoder as illustrated in Figure 3. The left branch from the first LSTM layer up was the encoder and the right was the decoder structure. Input was repeated after the first LSTM layer to enter the encoder and decoder layer, respectively. We defined a predict layer and tied all these together to establish the models. The basic LSTM layer was used for each encoder and decoder with 100 neurons and RELU activation. We compared the performances of losses, and the training time with five different optimizers (‘Adadelta’, “Adagrad”, “Adam”, “RMSprop” and “SGD”) using the same loss function (mean_squared_error) and learning rate. The test was repeated fifty times with the same parameters to avoid occasional bias.

**Figure 4:**
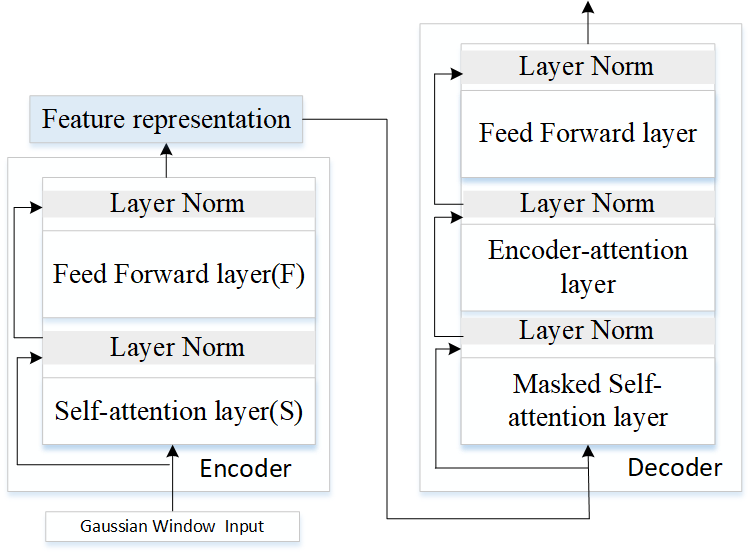
Transformers with attention mechanisms.

Inspired by the “sandwich transformer” of reordering the sublayers of more self-attention (S) toward the bottom and more feedforward (F) toward the top perform better [44], we designed the balanced architecture, which consist of four self-attention sublayers, four feed forward sublayers and reordered them from “FSFSFSFS” to “SSFSFSFF”. The proposed architecture of attention (Figure 4) was based on autoencoder framework with S layers and F layers as the encoder, and masked S, F, and encoder-attention layer as the decoder.

**Figure 5:**
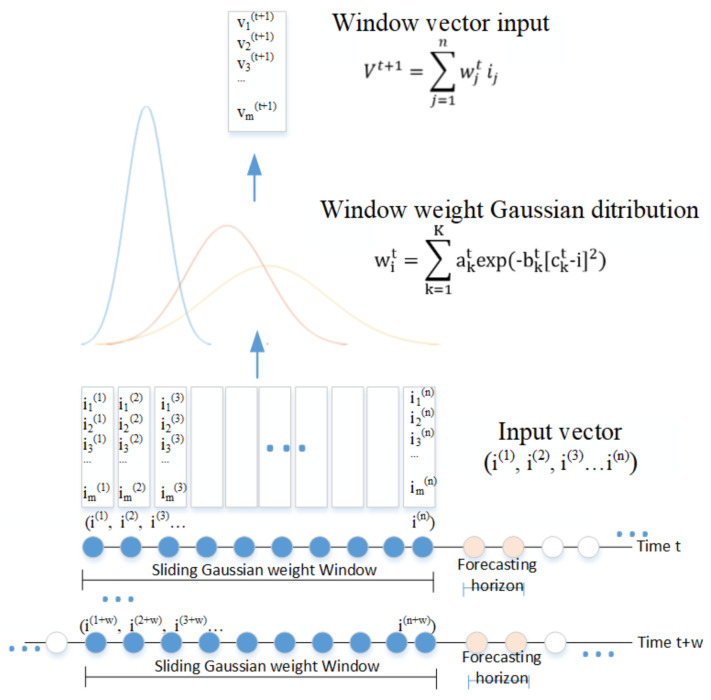
Gaussian sliding windows for initial weights in attention NN.

We proposed using the sliding Gaussian windows as the initial weights for the attention layer (Figure 5). The length of input vectors was n and the weights were randomly allocated for basic attention. Gaussian distribution for the weights was based on that distant part of the sequence might play a decreasing role. The parameters of $a_{k}$, $b_{k}$ and $c_{k}$ were optimized gradually using back propagation through time during training. The Gaussian windows vectors were then input into the LSTM with attention network demonstrated in Figure 5. Layer normalization reduced the “covariance shift” problems faced by batch normalization through fixing the mean and variance of the summed inputs within each layer [45]. We designed one RNN layer normalization between S and F layer.

Activation function of “Sigmoid” and “Tanh” were used in the basic LSTM cells (see Equation (1)). The basic function $\mathrm{softmax}\left(x_{i}\right)=e^{x_{i}}/\sum_{j}e^{x_{j}}$ might suffer from computational underflow (the value of numerator was very small) or overflow (the value of denominator was approaching zero). We proposed using the following equation $\mathrm{softmax}\left(\tilde{x_{i}}\right)=e^{x_{i}-max\left(x_{i}\right)}/\sum_{j}e^{x_{j}-max\left(x_{i}\right)}$ The value of denominator was at least more than $e^{0}=1$ and the numerator becomes larger so this problem was avoided. The optimizers selection influenced the training processes and the results, so five candidate optimizers were tested to find the most suitable one for the proposed models and dataset. Performances of losses, mean squared error rate and the training time were reported.

### 2.4. Temporal Convolutional Network Architectures for Forecasting and Classification

**Figure 6:**
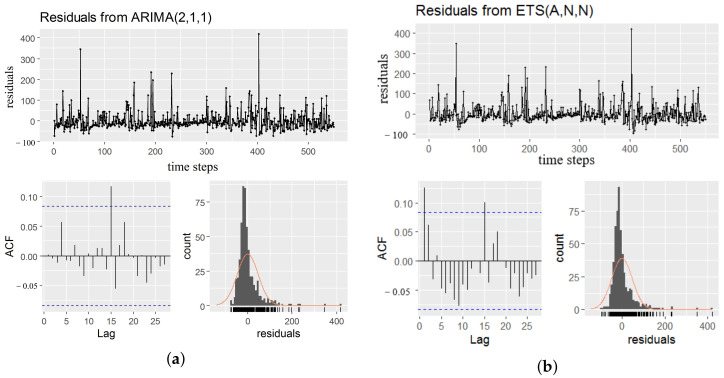
Comparisons between ARIMA and ETS models (**a**): ARIMA model, (**b**): ETS model.

TCN model with dilations was proposed for time series forecasting and classification task. TCN uses a 1D-FCN architecture where each hidden layer is the same length as the input layer, and zero padding of length is added to keep subsequent layers the same length. Causal convolutions where an output at time t is convolved only with elements from time t and earlier in the previous layer. Those features differentiate TCN architectures from FCN and CNN models. We illustrated the TCN models with dilation rate set to 4 and filter size to 3 in Figure 6. The number of CNN layers (convolutional 1D + activation + max_pooling) defined the TCN model architecture. To make the training process stable and achieve better performance fine-tuning the parameters of filter size, kernel size, pooling_size and dropout rate were crucial. The number of hidden CNN layers depends on the characteristic of the targeted data, which has no golden-rules and trial and error method were often used. We observed the dataset and constructed the TCN model with the number of hidden CNN layers set to 2, 3, 4, 5 which correlated with the filter size length. The flatten and dense layers were then added for the convolutional layers. The filter size, kernel size, pooling_size and drop out rate are all hyperparameters, which play important roles for the models’ performances. We proposed the grid search method and using the RMSE score to find the best fit parameters’ values. These four parameters, namely, dilation rate of [8,16,32,64], filter size of [16,32,64,128,256], kernel size of [2,3,4,5], pooling_size of [2,3,4,5] and dropout rate of [10%,20%,40%,60%], were set for the tests, which added up to 1280 (4×5×4×4×4) possible combinations. The candidate parameters which led to the unstable training process was deleted. For each of the combinations five optimizers were tested. The RMSE score was used to measure the performance. The combination of dilation rate of 64, filter size of 128, kernel size to 5, pooling_size to 2 and dropout rate to 20% for the regularization was chose for the model’s parameters.

### 2.5. Comparisons with Statistical and Machine Learning Methods

**Figure 7:**
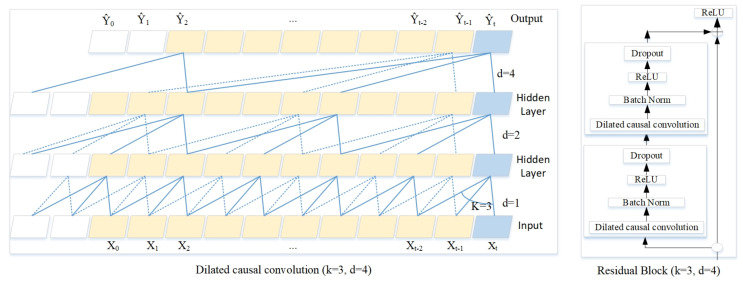
Dilated causal convolution with dilation rate d = 4, filter size k = 3 and residual block.

We constructed the statistical ARIMA and Exponential Smoothing (ETS) model to compare the performance with the proposed TCN models. Parameter p for AR, q for MA and d for difference orders determine the ARIMA model. For example ARIMA(2,1,3) means after processing of one order differencing, combines AR(2) model and MA(3). There are best practices for setting the values of these parameters such as observing the (partial) autocorrelation of the dataset, identifying the stationarity with differencing and the model selection (AR, MA, ARMA or ARIMA model), etc. After the model was selected, the parameters estimation process began. We followed the steps to construct two candidate models, compared the accuracy using two different parameter of p,d,q set to (2,1,2) and (3,0,0) for the same Kaggle web traffic dataset. Metrics showed ARIMA(2,1,1) was slightly better than ARIMA(3,0,0). ETS can be simplified as additive (A) or multiplicative (M) model. As for the target dataset, the additive model was more appropriate. We modeled the time series with ETS (A,N,N) (simple exponential smoothing with additive errors) and calculated the criteria. The analysis results of these two kinds of models were illustrated in Figure 7.

**Figure 8:**
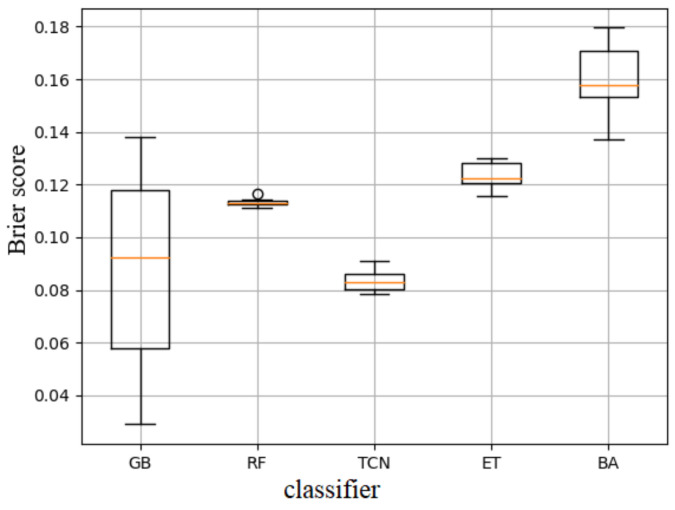
Performances comparisons among five models for time series classification task.

We compared performances of four classical machine learning classification algorithms which are Random Forest (RF), Gradient-Boosting tree (GB), Extra tree (ET) and Bagging methods (BA) with the proposed TCN model using the VPN-nonVPN dataset with the Brier scores in Figure 8. The Brier score (vertical axis in Figure 8) measures the accuracy of a probability forecast ranging from 0 to 1. $BS=\frac{1}{N}\sum_{t=1}^{N}\left(f_{t}-o_{t}\right)$, where $f_{t}$ is the forecast probability, $o_{t}$ is the outcome. The score of 0 stands for complete accuracy and 1 means the forecast was totally inaccurate. The results were illustrated using box-plot from which we concluded TCN model outperformed the others. Although GB method might perform better than TCN in some cases, the mean and the range of the scores was below the TCN model.

### 2.6. Generative Adversarial Architecture for Forecasting

**Figure 9:**
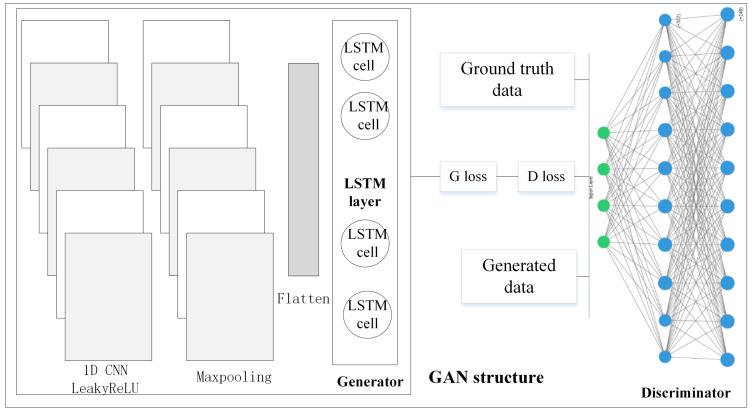
GAN structure for time series forecasting task.

The proposed GAN model was designed with the MLP as the *G* and the stacked LSTM as the *D* was for the time series forecasting task. The structures of *G* and *D* were given in Figure 9. We trained the model on the same web traffic dataset for 10,000 epochs with batch size set to 500 using the five optimizers (‘Adadelta’, ‘Adagrad’, ‘Adam’, ‘RMSprop’, and ‘SGD’), respectively.

## 3. Results and Discussion

The metric of loss we used for all the experiments was Mean Squared Error. Forecasting error for data *i*: $e_{i}=x_{i}-{\hat{x}}_{i}$, where ${\hat{x}}_{i}$ represents the forecast data *i*. SSE stands for sum of squared error and RMSE root mean squared error. The common metrics were defined in Equation (6) and used for TCN model designed for time series classification task. P, N, TP, FP, TN, FN stand for positive, negative, true positive, false positive, true negative, and false negative, respectively. For the optimizer of Adam, we set the parameters [46] of the algorithm: step size α=0.01, exponential decay rates for the first and second moment estimates $\beta_{1}=0.9$, $\beta_{2}=0.98$, and $\epsilon =10^{-6}$.

$$SSE=\sum_{i=1}^{n}e_{i}^{2}$$

$$RMSE=\sqrt{\frac{1}{n}SSE}$$

(6) $$\mathrm{Accuracy}=\frac{\mathrm{TP}+\mathrm{TN}}{P+N}=\frac{\mathrm{TP}+\mathrm{TN}}{\mathrm{TP}+\mathrm{TN}+\mathrm{FP}+\mathrm{FN}}\;$$

### 3.1. Long Short-Term Memory with Autoencoder

We used the same web traffic dataset and metric to evaluate the performances for this model.

We trained 100 epochs for each of five optimizers for fifty times. The results suggested that around 10 min per optimizer and the optimizer of ‘Adam’ and ‘Adagrad’ performed best with nearly the same RMSE score. Although the autoencoder model was more complex than the vanilla LSTM, we found the time required for training was only increased by less than 2 min and the RMSE scores slightly decreased.

### 3.2. Attention-Based Long Short-Term Memory

**Figure 10:**
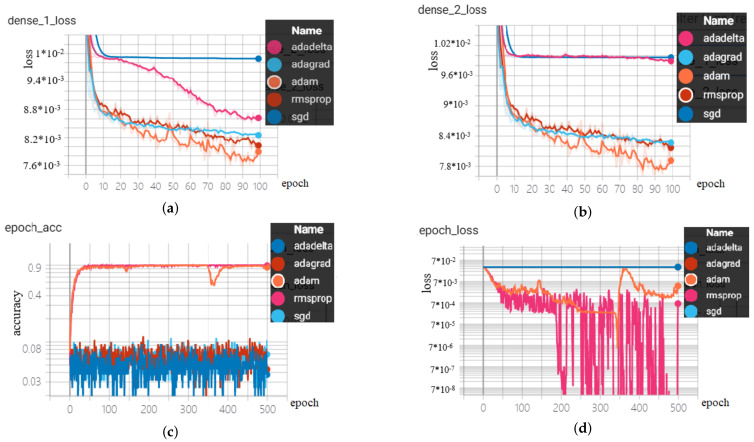
LSTM with autoencoder test results for time series forecasting task (**a**): encoder’s loss, (**b**): decoder’s loss, LSTM with Attention test (**c**): accuracy, (**d**): loss.

Kaggle web traffic dataset was used to evaluate the performances for this model. We trained 100 epochs for each optimizer for fifty times and found that training time was around 2 minutes per optimizer and the best performed optimizer was Adam. This model improved the performances by cutting training time by around 80 percent compared to vanilla LSTM and the error rate of RMSE also decreased (See Figure 10 and Table 1 for the results).

### 3.3. Temporal Convolutional Network

**Figure 11:**
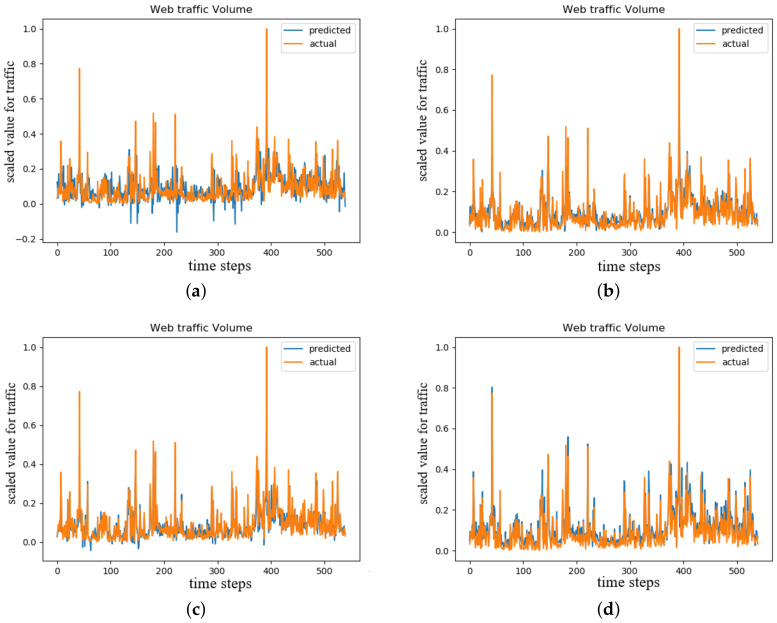
TCN with dilations test results for time series forecasting task (**a**) adadelta, (**b**) adagrad, (**c**) adam, (**d**) rmsprop.

The TCN model for time series forecasting task used the same web traffic dataset as we did in the LSTM autoencoder and attention mechanism experiments. Training time for TCN was the least among all the models with less than 1 min per 100 epochs, and the accuracy also improved compared to vanilla LSTM (see Figure 11). The second TCN model was for time series classification task of classifying of multi-variate, multi-class time series using the ECG dataset which achieved 99.5 percent of accuracy (see Table 2).

The tests showed different optimizers impacted significantly in the performances of accuracy and loss. The overall training time for each optimizer was around 3 min for 500 epochs (less than one minute for 100 epochs in Table 1).

**Figure 12:**
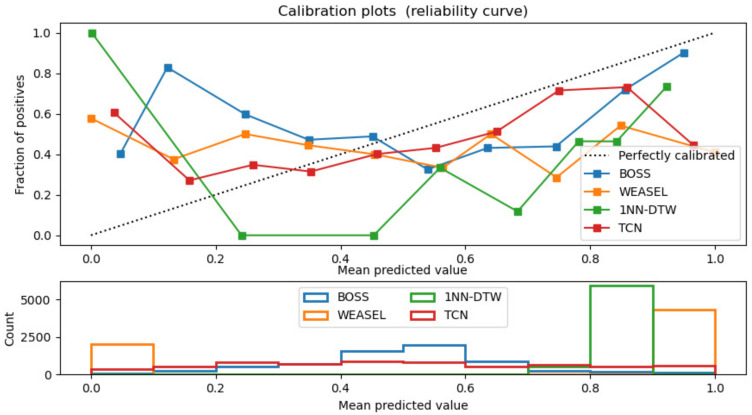
Models calibration comparisons among 1DNN-DTW, BOSS, WEASEL and TCN.

The best performed optimizer was ‘RMSprop’ which improved the loss rate to an order of magnitude. TCN’s efficiency was the best among the proposed models. The performance of TCN model for time series classification task was compared with the classical models of 1NN-DTW, WEASEL and BOSS using the VPN-nonVPN dataset. From the calibration curve in Figure 12 we could see that TCN was closest to the perfectly calibrated dashed line which indicated the proposed TCN model outperformed the aforementioned models.

### 3.4. Generative Adversarial Network

**Figure 13:**
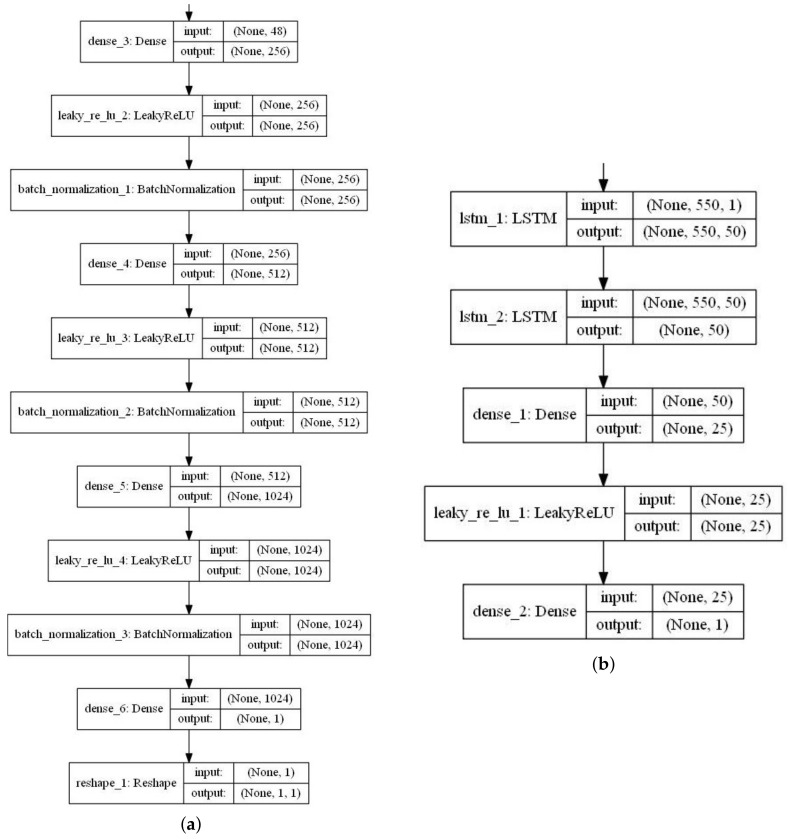
GAN structure for time series forecasting task (**a**): Generator (**b**): discriminator.

A GAN model for time series forecasting was proposed to model the Kaggle web traffic. The 500-time steps were used for training and the remaining 50-time steps for test. We set the same parameters (epochs, learning rate) for the models and loss functions as mean_squared_error for all the time series forecasting experiments. The detailed parameters were shown in Figure 13.

**Figure 14:**
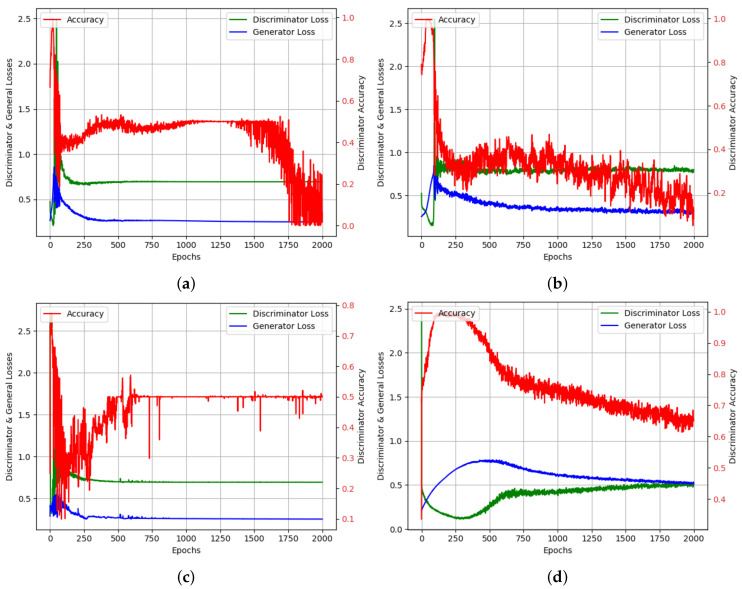
GAN test results for time series forecasting task loss and accuracy for Discriminator and Generator, (**a**) ‘adadelta’, (**b**) ‘adam’, (**c**) ‘rmsprop’, (**d**) ‘sgd’.

The same web traffic was used as the input to this GAN model. After 2000 epochs of training we plotted the results of the generator loss (blue curve), discriminator loss (green curve) and the discriminator accuracy (red curve) for different optimizers in Figure 14. The discriminator accuracy converged for optimizer of “Adadelta” and “Adam”. When using optimizer of “RMSprop” (c) and “SGD” (d), the discriminator accuracy arrived at around 50%, which meant that it had a relative high success rate of distinguishing the real dataset from generated ones.

This finding confirmed that training processes of GAN sometimes cannot converge to the satisfying minimum. Carefully choosing optimizers such as “Adam” and fine-tuned the corresponding parameters, the accuracy of discriminator converged to the minimum (see Figure 14).

## 4. Discussions

A challenge faced by LSTM is that it is unable to parallel. RNNs once became the dominant network architecture for translation and language modeling. However, one word at a time makes RNNs unable to parallel, so performance improvement became unavailable. This sequential nature also makes it hard to take advantage of fast computing devices such as TPU and GPU. Simply stacking the FCN, CNN and RNN or tweaking their parameters to get better performance for sequence were not enough and sometimes even failed. New architectures such as attention-based models (TCN and transformer) were then invented to increase performance, extract better features from data, generalization capability and reduce parameters.

TCN models with attention mechanisms have achieved remarkable successes, which makes us rethink RNN models’ situation in sequence modeling. Time complexity was analyzed to explain the experiment results why TCN outperformed others in terms of efficiency. In sequence models, the hidden state matrix is of size $d^{2}$ where *d* is the dimension of hidden state and *n* is the length of the input sentence. The cost of computing an input sentence of length *n* equals $n\times d^{2}$. The complexity of a sequence model where *d* and *n* are equal to 1000 and 50, respectively, is 50×1000×1000. For models based on attention mechanisms, the complexity is $n^{2}\times d$. This is 50×50×1000. This is the key reason that attention-based models are superior to the sequence model family ( RNNs / LSTMs)—the $n^{2}$ can be learned faster than the $d^{2}$ matrix- thus no need to sequentially back-propagate the errors through time. The experiments also confirmed the conclusions that TCN model has greatly improved the efficiencies. Attention models were fast growing field and many improvement solutions were put forward such as Sandwich transformer [44], Universal transformer [47], and the Residual shuffle exchange network [48] which requires less parameter compared to other models for the same task. Applying state of the art language models such as BERT, ELMO, GPT, etc. to the time series forecasting and classification areas worth researching.

The unstable training processes and lack of evaluation metrics hinder further development of GAN. Many academic papers on how to enable the stable training process were published, however the problem still needs further research. The evaluation metrics are still scarce (FID and IS dedicated for images quality) and lack of objectivity (judged by humans). Furthermore the majority of influential GAN papers were devoted their GAN models in the specific area of images, several for video generation, however the metrics for generation and forecasting of times series with GAN are much less (most cases visually, or RMSE metrics).

## 5. Conclusions

We proposed LSTM with autoencoder and attention, TCN with attention and GAN model to time series forecasting and classification, and all of these models accomplished the tasks. The performances of models using five different optimizers with the public datasets of Kaggle web traffic, VPN, ECG and the stress tests were conducted. For the time series classification task, the TCN model outperformed the classical algorithms such as random forest, gradient boosting and extra trees and bagging, and the GAN achieved comparable performances with statistical models such as ARIMA. We proposed the Gaussian sliding window weights to the attention mechanisms which showed reducing the training time greatly at least 80% while remained the same accuracy. “Adam” performed best among all five optimizers most of the time and the discretionary chosen parameters could result in the failure of convergence, especially for GAN models. The grid search method was employed in the experiments to the parameters tweaking, which helped the models’ reaching convergence and performed better.

## Figures and Tables

**Table 1:** Test results of web traffic forecasting with different optimizers ‘training/test losses (time)’.

Optimizer	LSTM	LSTM-auto	LSTM-att	TCN
Adadelta	57.98/49.23 (10 m 34 s)	51.57/39.34 (12 m 30 s)	46.77/40.12 (1 m 50 s)	49.34/40.15 (41 s)
Adagrad	59.21/51.34 (11 m 02 s)	45.74/40.83 (12 m 29 s)	46.77/41.29 (1 m 48 s)	46.24/39.94 (42 s)
Adam	51.20/46.91 (10 m 29 s)	45.21/40.60 (11 m 53 s)	45.12/40.40 (1 m 29 s)	45.82/39.61 (31 s)
Rmsprop	55.23/51.29 (11 m 38 s)	45.81/40.38 (13 m 42 s)	46.34/40.52 (1 m 39 s)	45.85/40.27 (45 s)
SGD	56.54/52.38 (11 m 44 s)	51.79/39.47 (13 m 36 s)	50.32/40.54 (1 m 52 s)	52.92/41.76 (44 s)

**Table 2:** Test results for different tasks with the proposed models.

Sequence Modeling	Model Size	LSTM	LSTM-auto	LSTM-Att	TCN
Adding problem (loss)	70 K	0.175	$6.2\times 10^{-3}$	$6.1\times 10^{-3}$	$5.9\times 10^{-3}$
MNIST (accuracy)	70 K	87.2	89.7	95.2	98.7
Music MIDI data (loss)	500 K	0.0822	0.0755	0.0671	0.0635
Copying memory (loss)	16 K	0.0301	0.0204	0.0198	0.0182
Kaggle web traffic (RMSE)	10 K	49.83	48.81	46.92	47.12
ECG classification (accuracy)	10 K	95.8	98.6	98.2	99.5

## Abbreviations

The following abbreviations are used in this manuscript:

MLP	Multiple Layer Perceptron
DNN	Deep Neural Network
TCN	Temporal Convolutional Networks
LSTM	Long Short-Term Memory
GAN	Generative Adversarial Network
RNN	Recurrent Neural Network
NLP	Natural Language Processing
ARIMA	Auto Regressive Integrated Moving Average
ETS	Exponential Smoothing
CNN	Covolutional Neural Network
ECG	Electrocardiogram
VPN	Virtual Private Network
FCN	Fully Connected Network
SSE	Sum of Squared Error
RMSE	Root Mean Squared Error
RF	Random Forest
GB	Gradient Boosting
ET	Extra Tree
BA	BAgging method
BS	Brier Score
RF	Generative Adversarial Network
